# Risk Factors and Outcomes of Vancomycin-Resistant *Enterococcus faecium* Bloodstream Infection in Hematologic Malignancies and Stem Cell Transplant Recipients: A Case-Control Study

**DOI:** 10.1093/ofid/ofag627

**Published:** 2026-10-06

**Authors:** Khuloud Aldhaheri, Jollee S T Fung, Cesilia Nishi, Jessica E Lau, Allison Mah, Alissa J Wright, Ryan J Stubbins, Marthe K Charles, Sara Belga

**Affiliations:** Department of Internal Medicine, College of Medicine and Health Sciences, United Arab Emirates University, Al Ain, Abu Dhabi, United Arab Emirates; Department of Medicine, Division of Infectious Diseases, University of British Columbia, Vancouver, British Columbia, Canada; Department of Medicine, Division of Infectious Diseases, University of British Columbia, Vancouver, British Columbia, Canada; Department of Pharmaceutical Sciences, Vancouver General Hospital, Vancouver, British Columbia, Canada; Faculty of Pharmaceutical Sciences, University of British Columbia, Vancouver, British Columbia, Canada; Department of Pharmaceutical Sciences, Vancouver General Hospital, Vancouver, British Columbia, Canada; Faculty of Pharmaceutical Sciences, University of British Columbia, Vancouver, British Columbia, Canada; Department of Medicine, Division of Infectious Diseases, University of British Columbia, Vancouver, British Columbia, Canada; Department of Medicine, Division of Infectious Diseases, University of British Columbia, Vancouver, British Columbia, Canada; Leukemia and Bone Marrow Transplant Program of British Columbia, BC Cancer, Vancouver, British Columbia, Canada; Department of Medicine, Division of Hematology, University of British Columbia, Vancouver, British Columbia, Canada; Division of Medical Microbiology and Infection Control, Department of Pathology and Laboratory Medicine, Vancouver General Hospital, Vancouver Coastal Health, Vancouver, British Columbia, Canada; Department of Pathology and Laboratory Medicine, University of British Columbia, Vancouver, British Columbia, Canada; Department of Medicine, Division of Infectious Diseases, University of British Columbia, Vancouver, British Columbia, Canada; Immunity and Infection Research Centre, Vancouver Coastal Health Research Institute, Vancouver, British Columbia, Canada

**Keywords:** bloodstream infection (BSI), cumulative antibiotic exposure, leukemia, stem cell transplant (SCT), vancomycin-resistant enterococci (VRE)

## Abstract

**Background:**

Vancomycin-resistant *Enterococcus faecium* (VRE*fm*) bloodstream infection (BSI) represents a growing challenge in hematology-oncology settings. This study aimed to characterize the epidemiology, risk factors, and prognostic impact of VRE*fm* BSI within a mixed adult hematology-oncology and transplant unit.

**Methods:**

We conducted a retrospective matched case-control study (2014–2024). Multivariable conditional logistic regression identified factors associated with VRE*fm* BSI, and restricted cubic spline (RCS) models assessed nonlinear exposure-response relationships between antimicrobial classes and VRE*fm* BSI. Survival was analyzed using Kaplan–Meier curves and Cox proportional hazards regression.

**Results:**

VRE*fm* BSI incidence rose from 0.02 to 0.07 per 1000 person-days (2014–2018 vs 2019–2024). A high-risk composite comprising recipients of allogeneic stem cell transplant and/or AML (adjusted OR [aOR], 3.90; 95% CI, 1.87–8.15; *P* < .001) and ICU admission (aOR, 5.63; 95% CI, 2.15–14.78; *P* < .001), were independently associated with VRE*fm* BSI. Cumulative days of carbapenems (aOR, 1.09/day; 95% CI, 1.02–1.17; *P* = .013), beta-lactam/beta-lactamase inhibitors (aOR, 1.09/day; 95% CI, 1.02–1.16; *P* = .006), and systemic antifungals (aOR, 1.12/day; 95% CI, 1.03–1.22; *P* = .009) exhibited duration-dependent associations. RCS models demonstrated a nonlinear, duration-dependent association with cumulative antibiotic exposure, including antianaerobic agents (*P* < .001). VRE*fm* BSI was associated with increased mortality (adjusted hazard ratio, 2.09; 95% CI, 1.63–2.66; *P* < .001).

**Conclusions:**

VRE*fm* BSI reflects the interplay between host susceptibility and nonlinear, duration- and spectrum-dependent antibiotic exposure pressure. This underscores the importance of antimicrobial stewardship strategies as a potential intervention to mitigate risk.

## Teaser

Among 3301 patients with hematological malignancy or a stem cell transplant, 100 cases with VRE*fm* BSI were matched to 400 controls. Factors associated with VRE*fm* BSI included cumulative exposure to beta-lactams and antifungals, underscoring stewardship as a preventive strategy.

Enterococci are facultative anaerobic, gram-positive cocci that constitute part of the normal human microbiota in the gastrointestinal, vaginal, and oral tracts [1]. Vancomycin resistance in clinical enterococci isolates was first reported in 1986 [2]. Since then, VRE has become a concern within healthcare settings, including ICUs and among patients with hematological malignancies [2, 3]. VRE is recognized as a significant nosocomial pathogen associated with severe healthcare-associated infections, including bloodstream infections (BSIs), which have higher mortality rates than infections caused by vancomycin-susceptible enterococci [4, 5]. Epidemiologically relevant VRE are *Enterococcus faecium* or *Enterococcus faecalis* that acquire *vanA* or *vanB* genes, conferring high-level vancomycin resistance [2, 6]. These pathogens colonize patients, persist within hospital environments, and spread through contact and surface contamination [5]. Beyond hospital ecology, multiple factors have contributed to the rising prevalence of VRE, including antibiotic selection pressure, invasive medical devices, a growing population of vulnerable patients, and potential gaps in stewardship and infection control [7, 8].

Hospitalized patients diagnosed with leukemia who are receiving chemotherapy, especially AML induction chemotherapy, and those undergoing stem cell transplant (SCT), are among the most vulnerable to VRE BSI [2, 9, 10]. Allogeneic SCT (allo-SCT) recipients face a higher burden of VRE BSI and increased mortality compared with autologous SCT (auto-SCT) recipients, who have lower mortality despite frequent colonization [6, 11, 12]. Patients diagnosed with AML exhibit intermediate risk, largely driven by gastrointestinal colonization and specific antibiotic exposures, particularly aminoglycosides, antianaerobic agents, and beta-lactams (BLs) [9, 13]. The increased risk within this population stems from significant antibiotic selection pressure and microbiome disruption, which can lead to intestinal VRE dominance before the onset of VRE BSI [13, 14]. Additionally, chemotherapy-induced mucosal barrier injury facilitates VRE translocation from the gut to the bloodstream, and prolonged neutropenia impairs innate defenses amid high VRE colonization pressure [2, 6, 12–16]. Further risk factors for VRE BSI within this cohort include the timing of VRE colonization, high unit transmission burden, renal dysfunction, the degree of immunosuppression, and hospital-related factors, specifically, the presence of central venous catheters, ICU admissions, and longer hospital stays [2]. The differing incidence, mortality, and risk profiles of VRE BSI across different cohorts necessitate tailored antimicrobial stewardship and infection prevention and control strategies in hematology-oncology and SCT units [6, 17].

While VRE BSI epidemiology is well characterized in certain populations, including patients undergoing SCT, evidence remains limited in mixed units that include patients with diverse treatment exposure, hematologic malignancies, and transplant histories [3, 18, 19]. Studies examining VRE BSI risk factors and outcomes across these cohorts, within the context of shared stewardship practices and local colonization pressure, remain sparse [7, 10, 20]. Moreover, previous studies have used binary models to analyze antimicrobial exposure, with limited investigation of how the cumulative duration and spectrum of exposure affect VRE BSI in these populations [21, 22]. To address these gaps, we aim to identify risk factors and clinical outcomes, including all-cause mortality, associated with VRE BSI among hospitalized patients with hematological malignancies or SCT recipients managed within a shared unit. We also aim to evaluate and characterize the cumulative effect of antibiotic exposure on VRE BSI in this population.

## METHODS

### Study Design

We conducted a retrospective, matched case-control study of adult patients (≥18 years) admitted to the leukemia and bone marrow transplant (LBMT) unit at Vancouver General Hospital, the province's referral center for the LBMT program. The study included patients admitted between January 1, 2014 and January 31, 2024. Data collected from electronic medical records (EMRs) included demographics, clinical information, microbiology results, and hospitalization details.

### Ethics

This study used retrospective data, handled in accordance with institutional privacy and research ethics requirements. Approval was granted by the University of British Columbia Research Ethics Board (H24-00362).

### Patient Population

The source population included all patients admitted to the LBMT unit during the study period. Cases were patients who developed vancomycin-resistant *Enterococcus faecium* (VRE*fm*) BSI during hospitalization, defined as isolation of *E. faecium* with a vancomycin MIC ≥32 μg/mL from at least 1 positive blood culture. Recurrent VRE*fm* BSI cases were counted only once at initial onset. We excluded patients who developed VRE*fm* BSI as outpatients. Operational definitions are provided in the Supplementary material. Controls, selected from the same source population, did not develop VRE*fm* BSI, and were matched to cases at a 1:4 ratio based on admission indication–assessment, chemotherapy, transplant, or complication–to control for immunosuppression and clinical acuity. Each indication is defined in the Supplementary material. Additionally, controls were matched to the admission period (within ± 3 months of the case's admission date) to account for secular trends in infection control policies and antimicrobial practices.

### Primary Exposures and Outcomes

The primary outcome was VRE*fm* BSI during the index hospitalization; the secondary outcome was all-cause mortality. The primary exposures included age, severe neutropenia, ICU admission, hospital length of stay (LOS), primary hematologic malignancy diagnosis, SCT type, and cumulative antibiotic class exposure during hospitalization; definitions of antimicrobial classes are outlined in the Supplementary material.

### Data Sources and Measurement

Data were retrospectively collected from EMR systems used during the study periods. For cases, the date of VRE*fm* BSI onset was considered the index date and time-at-risk was calculated from the date of hospital admission to this date. To align time-at-risk periods, controls were assigned the corresponding case's index date, thereby using the same interval from their admission date, when feasible. For controls discharged before the matched case's index date, time-at-risk was calculated from admission to discharge.

### Variable Selection

We selected variables a priori based on clinical plausibility and established risk factors for VRE BSI in the hematology-oncology and SCT literature, including ICU admission, severe neutropenia at any time during hospitalization, SCT type, primary hematological malignancy diagnosis, and cumulative antibiotic class exposure. Severe neutropenia was defined as an absolute neutrophil count <0.5 × 10^9^/L. We measured antibiotic class exposure as the total number of days of therapy for each antibiotic class, including fluoroquinolones, carbapenems, cephalosporins, BL/beta-lactamase inhibitors (BL/BLIs), vancomycin, antianaerobic agents, and systemic antifungals. We defined antimicrobial exposure for cases as the total days of therapy from admission to the index date. For matched controls, we calculated exposure from admission to the matched index date, or to discharge if discharge occurred earlier. We selected this hospital admission-based timeline approach to evaluate the cohort within a consistently defined hospitalization and risk period. We recorded VRE colonization status when feasible; however, routine weekly prevalence screening was discontinued in March 2018, limiting data availability during the latter study period. Other variables considered but excluded were graft-versus-host disease (GVHD), GVHD type and treatment due to missing data and nonapplicability to all participants in this mixed LBMT unit. We also excluded primary disease status and staging, cumulative chemotherapy received, immunosuppression therapy, mucositis, and acute kidney injury due to incomplete EMR data.

### Statistical Analysis

The overall incidence rate of VRE*fm* BSI was calculated as the number of cases per 1000 patient-days at risk, stratified by era (2014–2018 vs 2019–2024). Baseline characteristics were summarized using descriptive statistics. Continuous variables were summarized as medians with IQRs, and compared using the Wilcoxon rank-sum test. Categorical variables were reported as frequencies and percentages and compared using the χ^2^ test or Fisher's exact test where cell counts were small.

#### Univariable and Multivariable Logistic Regression Analysis

We used conditional logistic regression in both univariable and multivariable analyses to account for the matched design and to estimate ORs, adjusted ORs (aORs), and 95% CIs. This method accounts for the 1:4 matched design by conditioning on the stratum-specific intercept (the matched set). The final multivariable model was prespecified and adjusted for the combined high-risk composite (AML and/or allo-SCT), severe neutropenia, ICU admission, and total days of antibiotic class use (fluoroquinolones, carbapenems, BL/BLIs, and systemic antifungals). We excluded antianaerobic agents from the multivariable model because the class definition overlapped significantly with carbapenems and BL/BLIs (Supplementary material). Two-sided *P*-values < .05 were considered statistically significant. We assessed multicollinearity among predictors using variance inflation factors (VIFs) adapted for conditional logistic regression.

#### Restricted Cubic Spline Analysis of Antibiotic Durations

To explore the nonlinear relationships between cumulative antibiotic class exposure and the odds of VRE*fm* BSI, we used restricted cubic spline (RCS) models. We measured cumulative exposure as observed inpatient antibiotic-days. We placed spline knots at 7, 14, and 21 days to reflect clinically meaningful durations of continuous therapy. We truncated the exposure range at 25 days for stability. We assessed RCS models separately for each antibiotic class to avoid collinearity and overfitting. We adjusted RCS models for high-risk composite, severe neutropenia, ICU admission, and hospital LOS. We used a large language model–based assistant (Microsoft Copilot) as a troubleshooting tool during the RCS analysis; we limited its use to decision support, and we independently reviewed and verified all outputs.

#### Sensitivity Analyses

We conducted 3 sensitivity analyses to evaluate the robustness of the primary multivariable model. First, we assessed the independent associations of primary diagnosis and SCT type by modeling these variables simultaneously. Second, we deconstructed the high-risk composite into its components (AML and allo-SCT) while retaining other prespecified variables. Third, to account for differences in antibiotic exposure opportunity, we standardized antibiotic exposure as antibiotic-days divided by the number of observed inpatient days before the index date (or discharge date for controls discharged earlier). This exposure-density analysis accounts for the variation in time at risk.

#### Survival and Mortality Outcomes Analysis

We evaluated the relationship between VRE*fm* BSI and mortality by comparing survival rates of patients with VRE*fm* BSI to those in the larger LBMT cohort during the study period (2014–2024). We analyzed survival data using univariable and multivariable Cox proportional hazards regression. Results are expressed as HRs and adjusted HRs (aHRs) with 95% CIs. Survival probabilities were also plotted using Kaplan–Meier curves. We censored patients at the last known follow-up or administratively on January 31, 2024.

We preformed analyses using Stata/SE (StataCorp LLC, version 19.5, College Station, TX).

## RESULTS

### Incidence Rates

Over the study period from January 1, 2014 to January 31, 2024, 3301 patients were hospitalized in the LBMT unit. The overall incidence rate of VRE*fm* BSI was 0.03 per 1000 person-days. The incidence rate increased over time, rising from 0.02 to 0.07 per 1000 person-days in 2014–2018 compared with 2019–2024. A total of 102 cases of VRE*fm* BSI were initially identified; 2 cases were excluded from the case-control analysis because the onset of VRE*fm* BSI occurred prior to the admission date. Among the 100 cases, 0.05% of participants (n = 5) experienced recurrent VRE*fm* BSI.

### Baseline Characteristics

The median age in the cohort (n = 500) was 56.5 years (IQR 42.5–64.0), and 61.6% were males. Significant differences were observed in the underlying diagnosis: patients with AML were overrepresented among cases compared with controls (54% vs 36.2%), whereas patients with non-Hodgkin lymphoma (NHL) were less frequent among cases than controls (7% vs 20.8%) (Figure 1). Regarding SCT type, cases were predominantly allo-SCT (36% vs 14.2%; *P* < .001), whereas almost all auto-SCT participants were controls (17% vs 1%; *P* < .001). Cases had a longer median hospital LOS compared with controls (42.5 days [IQR, 29.5–60.5] vs 22.0 days [IQR, 10.0–30.0]; *P* < .001), and overall, a more critically ill cohort with 41% of cases requiring ICU admission compared with 5% in controls (*P* < .001). Most cases had severe neutropenia, whereas fewer controls experienced neutropenia during their hospital stay (98% vs 79.5%; *P* < .001). Except for cephalosporins, cumulative antimicrobial exposure was significantly higher in cases. Specifically, cases had a greater median duration exposure to BL/BLIs (9.0 [IQR, 3.0–16.0] vs 5.0 [IQR, 1.0–10.0] days; *P* < .001), carbapenems (2.0 [IQR, 0–9.0] vs 0 [IQR, 0–1.5] days; *P* < .001), antianaerobic agents (14.5 [IQR, 8.0–24.0] vs 7.0 [IQR, 3.0–13.0] days; *P* < .001), vancomycin (2.0 [IQR, 0–6.0] vs 0 [IQR, 0–2] days; *P* < .001), and systemic antifungals (15.0 [IQR, 10.0–22.0]) versus (10.0 [IQR, 3.0–15.0]; *P* < .001). Furthermore, a higher proportion of cases than controls were exposed to fluoroquinolones (16% vs 8.5%; *P* = .025). Notably, clinical suspicion of infection remained elevated among controls: 82.8% were exposed to antianaerobic agents, 78.2% to systemic antifungals, 28.7% to vancomycin, and 26.2% to carbapenems (Table 1).

**Figure 1.**
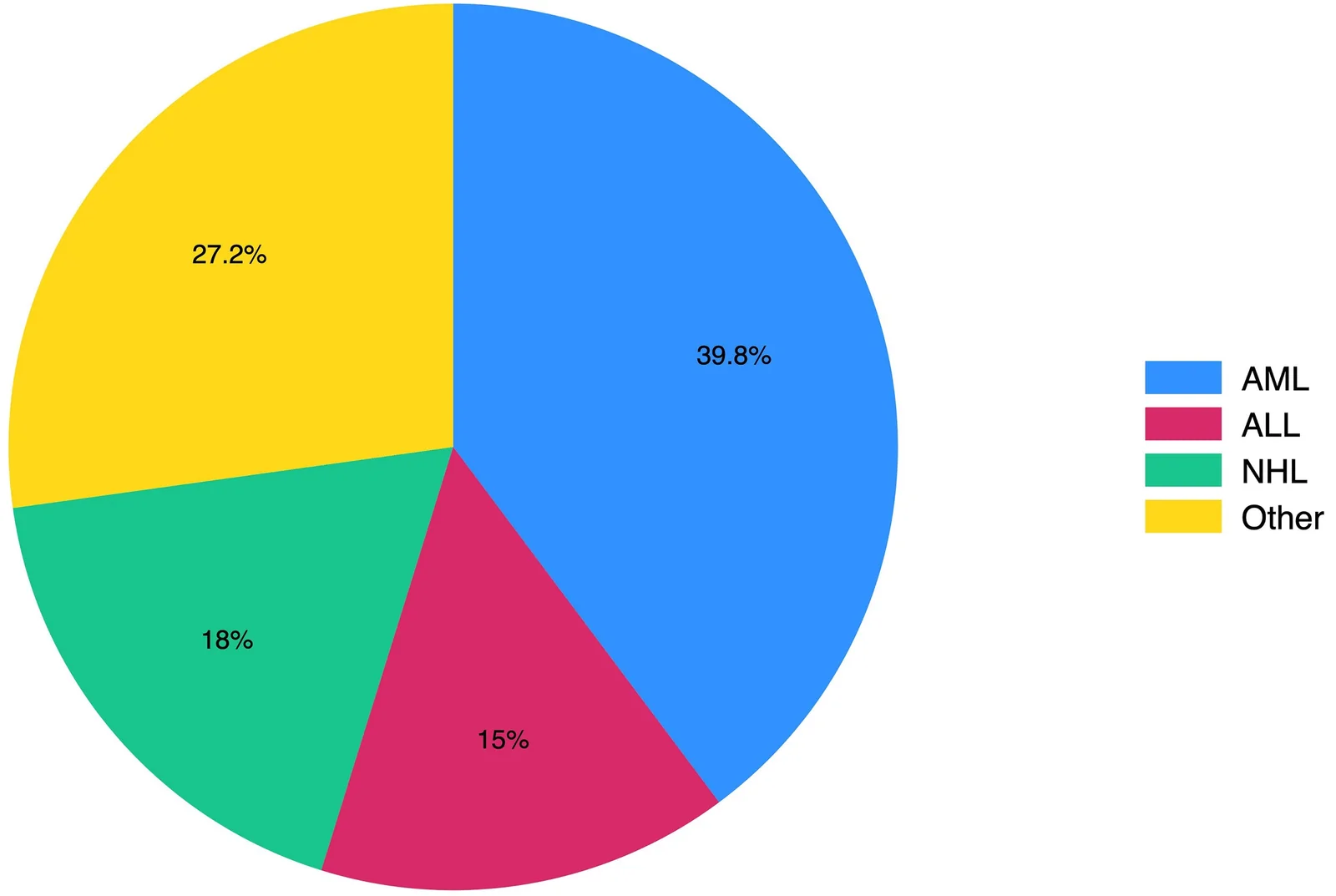
Distribution of primary hematologic diagnoses among all participants in the matched case-control cohort (n = 500). Abbreviations: VRE*fm*, vancomycin-resistant *Enterococcus faecium*; BSI, bloodstream infection; AML, acute myeloid leukemia; ALL, acute lymphoblastic leukemia; NHL, non-Hodgkin lymphoma.

**Table 1.** Baseline Characteristics of VRE*fm* BSI Cases and Matched Controls in the Matched Case-control Population (n = 500)

Characteristic, N (%)	Total (n = 500)	Controls (n = 400)	Cases (n = 100)	*P* Value
Age, median (IQR)	56.5 (42.5–64.0)	57.0 (42.0–64.0)	55.0 (46.0–62.0)	.420
Male	308 (61.6)	251 (62.7)	57 (57.0)	.290
Primary hematological malignancy diagnosis, N (%)				
AML	199 (39.8)	145 (36.2)	54 (54.0)	.002
ALL	75 (15.0)	57 (14.2)	18 (18.0)	
NHL	90 (18.0)	83 (20.8)	7 (7.0)	
HL	15 (3.0)	13 (3.2)	2 (2.0)	
MPD/MDS	53 (10.6)	40 (10.0)	13 (13.3)	
Other leukemia	12 (2.4)	10 (2.5)	2 (2.0)	
Aplastic anemia	16 (3.2)	13 (2.3)	3 (3.0)	
Multiple myeloma	32 (6.4)	32 (8.0)	0	
Other	8 (1.6)	7 (1.6)	1 (1.0)	
Transplant status, N (%)				
Allogeneic SCT	93 (18.6)	57 (14.2)	36 (36.0)	<.001
Autologous SCT	69 (13.8)	68 (17.0)	1 (1.0)	<.001
Clinical variables, median days (IQR) for continuous, N (%) for categorical				
Hospital LOS	25.0 (14.5–34.0)	22.0 (10.0–30.0)	42.5 (29.5–60.5)	<.001
Severe neutropenia^a^	416 (83.2)	318 (79.5)	98 (98.0)	<.001
ICU admission^b^	61 (12.2)	20 (5.0)	41 (41.0)	<.001
VRE colonization^c^	42 (8.4)	24 (6.0)	18 (18.0)	<.001
Cumulative antimicrobials, median days (IQR)				
BL/BLIs	6.0 (2.0–11.0)	5 (1.0–10.0)	9.0 (3.0–16.0)	<.001
Cephalosporins	0.0 (0.0–0.0)	0.0 (0.0–0.0)	0.0 (0.0–0.0)	.130
Carbapenems	0.0 (0.0–4.0)	0.0 (0.0–1.5)	2.0 (0.0–9.0)	<.001
Antianaerobic agents	8.0 (4.0–15.5)	7.0 (3.0–13.0)	14.5 (8.0–24.0)	<.001
Vancomycin	0.0 (0.0–3.0)	0.0 (0.0–2.0)	2.0 (0.0–6.0)	<.001
Fluoroquinolones	0.0 (0.0–0.0)	0.0 (0.0–0.0)	0 (0.0–0.0)	<.001
Systemic antifungals	10.0 (4.0–16.0)	10.0 (3.0–15.0)	15.0 (10.0–22.0)	<.001
Exposure to antimicrobials,^d^ N (%)				
BL/BLIs	87 (17.4)	56 (14.0)	31 (31.0)	<.001
Cephalosporins^e^	80 (16.0)	59 (14.8)	21 (21.0)	.130
Carbapenems	167 (33.4)	105 (26.2)	62 (62.0)	<.001
Antianaerobic agents	427 (85.4)	331 (82.8)	96 (96.0)	<.001
Vancomycin	171 (34.2)	115 (28.7)	56 (56.0)	<.001
Fluoroquinolones	50 (10.0)	34 (8.5)	16 (16.0)	.025
Systemic antifungals	410 (82.0)	313 (78.2)	97 (97.0)	<.001

Median IQR for continuous variables and N (%) for categorical variables.

Abbreviations: VRE*fm*, vancomycin-resistant *Enterococcus faecium*; BSI, bloodstream infection; IQR, interquartile range; AML, acute myeloid leukemia; ALL, acute lymphoblastic leukemia; HL, Hodgkin lymphoma; NHL, non-Hodgkin lymphoma; MPD, myeloproliferative disorder; MDS, myelodysplastic syndrome; SCT, stem cell transplant; CAR-T, chimeric antigen receptor T cell therapy; ICU, intensive care unit; LOS, length of stay; BL/BLIs, beta-lactam/beta-lactamase inhibitors.

^a^Absolute neutrophil count <0.5 × 10^9^/L at any point during index hospitalization.

^b^Admission to ICU during index hospitalization at any point.

^c^VRE surveillance was discontinued in March 2018; values are missing for 294 controls 294 and 73 cases.

^d^All antimicrobials were prescribed for ≥14 d except cephalosporins.

^e^Cephalosporins prescribed ≥1 d.

### Univariable and Multivariable Analyses of Factors Associated With VRE*fm* BSI

In univariable analysis, factors associated with VRE*fm* BSI included high-risk composite, defined as a diagnosis of AML and/or recipient of allo-SCT (OR, 4.38; 95% CI, 2.60–7.41; *P* < .001), hospital LOS (OR, 1.05; 95% CI, 1.03–1.06; *P* < .001), severe neutropenia (OR, 14.23; 95% CI, 3.39–59.78; *P* < .001), ICU admission (OR, 14.14; 95% CI, 7.04–28.38; *P* < .001), and the following cumulative antibiotic exposure: carbapenems (OR, 1.13; 95% CI, 1.08–1.17; *P* < .001), BL/BLIs (OR, 1.10; 95% CI, 1.06–1.14; *P* < .001), vancomycin (OR, 1.14; 95% CI, 1.08–1.20; *P* < .001), fluoroquinolones (OR, 1.16; 95% CI, 1.07–1.25; *P* < .001), antianaerobic agents (OR, 1.14; 95% CI, 1.10–1.18; *P* < .001), and systemic antifungals (OR, 1.22; 95% CI, 1.14–1.30; *P* < .001) (Table 2).

**Table 2.** Univariable and Multivariable Analyses of Factors Associated With VRE*fm* BSI in the Matched Case-control Population (n = 500)

Variable	Crude OR (95% CI)	*P* Value	Adjusted OR (95% CI)	*P* Value
Age	1.00 (0.98–1.01)	.865	…	…
High-risk composite^a^	4.38 (2.60–7.41)	<.001	3.90 (1.87–8.15)	<.001
Hospital length of stay	1.05 (1.03–1.06)	<.001	1.01 (.99–1.02)	.248
Severe neutropenia	14.23 (3.39–59.78)	<.001	6.13 (.91–41.38)	.063
ICU admission	14.14 (7.04–28.38)	<.001	5.63 (2.15–14.78)	<.001
Carbapenems	1.13 (1.08–1.17)	<.001	1.09 (1.02–1.17)	.013
BL/BLIs	1.10 (1.06–1.14)	<.001	1.09 (1.02–1.16)	.006
Vancomycin	1.14 (1.08–1.20)	<.001	0.94 (.86–1.03)	.214
Fluoroquinolone	1.16 (1.07–1.25)	<.001	1.17 (.98–1.41)	.083
Antianaerobic agents	1.14 (1.10–1.18)	<.001		
Systemic antifungals	1.22 (1.14–1.30)	<.001	1.12 (1.03–1.22)	.009

Abbreviations: VRE*fm*, vancomycin-resistant *Enterococcus faecium*; BSI, bloodstream infection; SCT, stem cell transplant; AML, acute myeloid leukemia; ALL, acute lymphoblastic leukemia; ICU, intensive care unit; BL/BLIs, beta-lactam/beta-lactamase inhibitors.

^a^High-risk composite: AML and/or allo-SCT regardless of indication.

In the multivariable model, high-risk composite (aOR, 3.90; 95% CI, 1.87–8.15; *P* < .001), ICU admission (aOR, 5.63; 95% CI, 2.15–14.78; *P* < .001), and cumulative exposure to several antimicrobial classes remained independently associated with VRE*fm* BSI. Specifically, the odds of VRE*fm* BSI increased for each additional day by: 9% for carbapenem (aOR, 1.09; 95% CI, 1.02–1.17; *P* = .013), 9% for BL/BLIs (aOR, 1.09; 95% CI, 1.02–1.16; *P* = .006), and 12% for systemic antifungals (aOR, 1.12; 95% CI, 1.03–1.22; *P* = .009). Cumulative vancomycin and fluoroquinolone exposures were not associated with VRE*fm* BSI after adjustment. The multivariable estimate of severe neutropenia was notably imprecise (aOR, 6.13; 95% CI, .91–41.38; *P* .063) due to data sparsity, with 98.0% of cases and only 79.5% of controls experiencing severe neutropenia (Table 2). The mean VIF was 1.71 (maximum 3.17), indicating that multicollinearity did not otherwise destabilize the multivariable model.

### RCS Analysis of Antibiotic Durations

RCS analyses identified a nonlinear association between VRE*fm* BSI and cumulative exposure to: carbapenems (*P* = .022) (Figure 2*A*), BL/BLIs (*P* = .026) (Figure 2*B*), antianaerobic agents (*P* < .001) (Figure 2*C*), and systemic antifungals (*P* = .001) (Figure 2*D*). The odds of VRE*fm* BSI progressively increased with cumulative antibiotic exposure. The analysis demonstrated an apparent rise at higher exposure durations, particularly beyond 15 days. However, the widening 95% CI limited precision in the upper exposure range, and no definitive exposure threshold could be established. The most notable acceleration was observed with systemic antifungals, whereas antianaerobic agents demonstrated the lowest cumulative exposure. There was no evidence of nonlinearity among vancomycin, fluoroquinolones, and cephalosporins.

**Figure 2.**
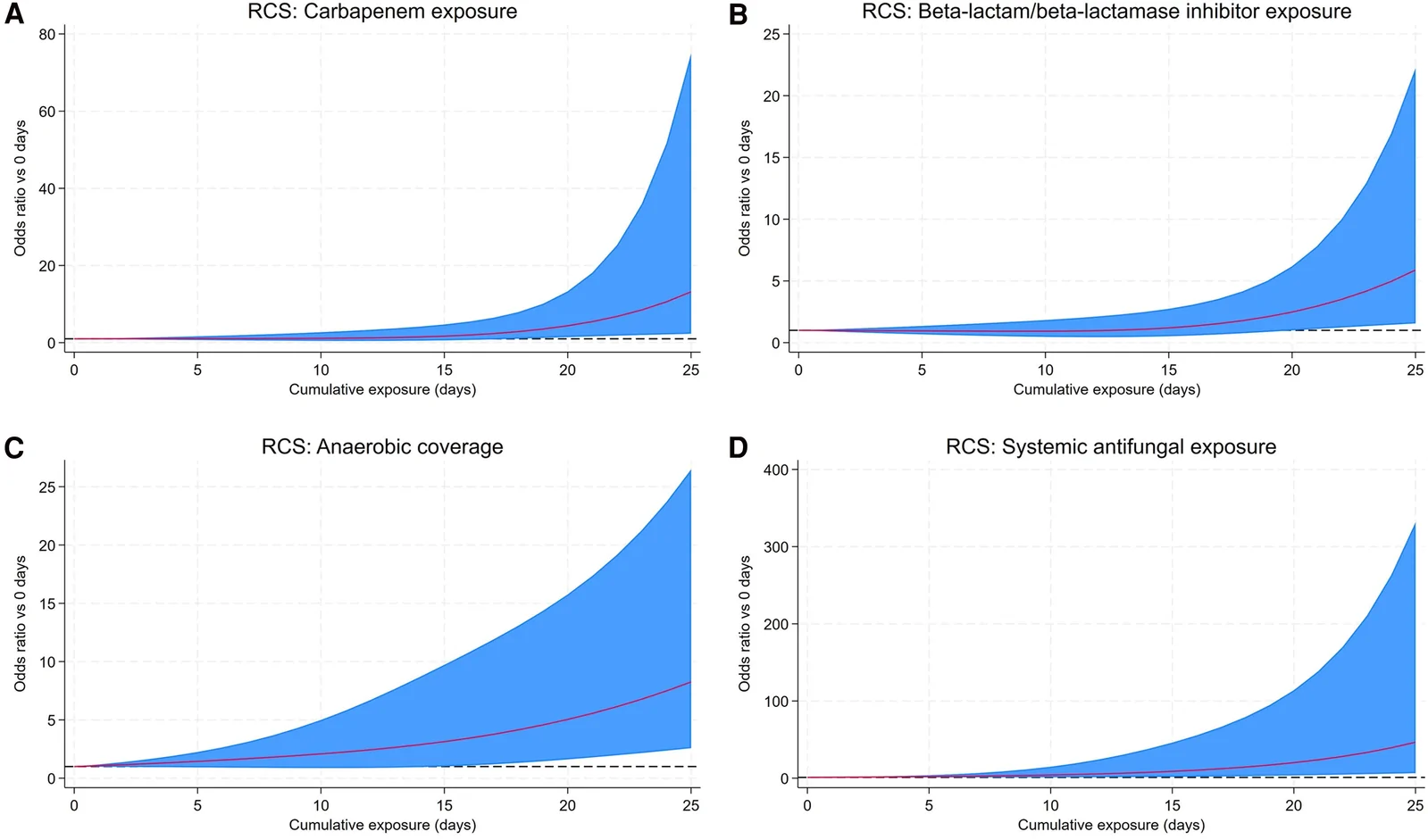
Restricted cubic spline models: The solid line represents the aORs for VRE*fm* BSI relative to day 0 of exposure, the dashed line represents the reference value of an odds ratio of 1, and the shaded band represents the 95% CI. (*A)* carbapenems (top left), (*B*) BL/BLIs (top right), (*C*) antianaerobic agents (bottom left), and (*D*) systemic antifungals (bottom right). Abbreviations: *VREfm*, vancomycin-resistant *Enterococcus faecium*; BSI, bloodstream infection; aORs, adjusted odds ratios; CI, confidence interval; BL/BLIs, beta-lactams/beta-lactamase inhibitors.

### Sensitivity Analyses

In the analysis evaluating the risk of VRE*fm* BSI associated with primary diagnosis and SCT type, only AML and allo-SCT were associated with VRE*fm* BSI, while other diagnoses and auto-SCT were not associated with VRE*fm* BSI (Supplementary Table 1). Supplementary Table 2 shows the distribution of SCT type by primary diagnosis.

When the high-risk composite variable was deconstructed into AML and allo-SCT; allo-SCT remained strongly associated with VRE*fm* BSI; however, AML was no longer statistically significant. The AML point estimate remained stable, but the aOR was 2.02 with a wider 95% CI than 1.99 in the primary model (Supplementary Table 1), Fluoroquinolone exposure became statistically significant in this analysis (Supplementary Table 3).

In the exposure-density analysis, high-risk composite, hospital LOS, and ICU admission remained associated with VRE*fm* BSI, whereas no antimicrobial class exposure was associated with VRE*fm* BSI. Severe neutropenia became statistically significant (Supplementary Table 4).

### Survival Following VRE*fm* BSI

In the full LBMT cohort (n = 3301), the all-cause mortality rate was 0.4 per 1000 person-days in 2014–2018 and 0.5 per 1000 person-days in 2019–2024. Mortality rates were higher among patients with VRE*fm* BSI than among those without VRE*fm* BSI in both periods, increasing from 0.7 versus 0.4 per 1000 person-days in 2014–2018 to 1.3 versus 0.5 per 1000 person-days in 2019–2024, respectively.

VRE*fm* BSI was associated with reduced 1-year overall survival in the overall cohort (*P* < .001; Figure 3). This survival divergence persisted over a 3-year follow-up period (*P* < .001; Supplementary Figure 1). Over the entire follow-up period, VRE*fm* BSI was associated with a significantly increased mortality in the unadjusted model (HR, 2.10; 95% CI, 1.65–2.69; *P* < .001) and remained significant after adjusting for age and sex (aHR, 2.09; 95% CI, 1.63–2.55; *P* < .001).

**Figure 3.**
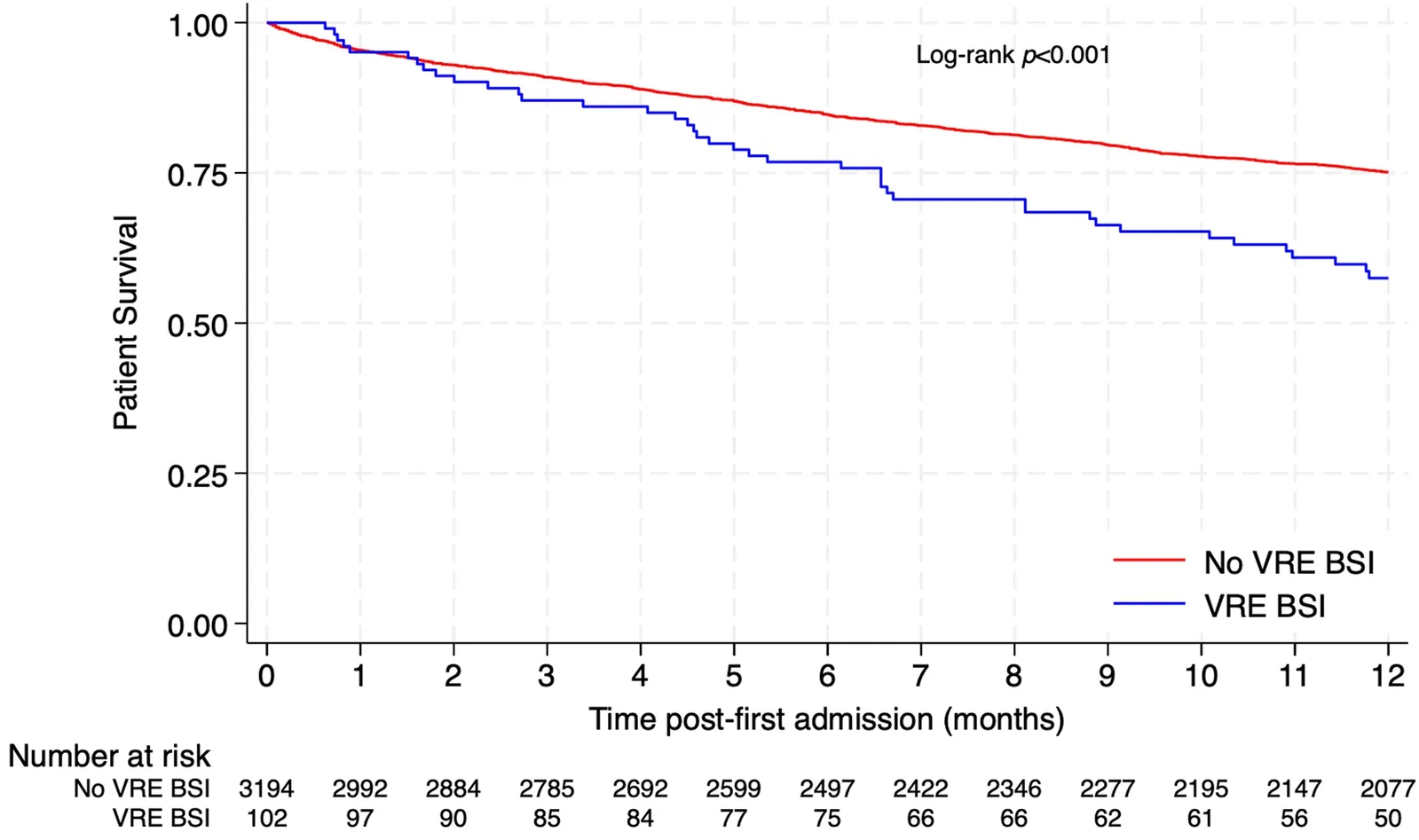
Kaplan–Meier survival curves for 1-year overall survival in the overall LBMT cohort (n = 3301). Abbreviations: VRE, vancomycin-resistant *Enterococcus faecium*; BSI, bloodstream infection; LBMT, leukemia and bone marrow transplant.

## DISCUSSION

In this large cohort of patients with leukemia and SCT managed within a shared mixed unit, we identified a high-risk composite for VRE*fm* BSI, comprising patients with AML and/or allo-SCT recipients. Over the study period, the incidence of VRE*fm* BSI increased more than 3-fold. Cumulative antimicrobial exposure showed a nonlinear, spectrum- and duration-dependent association with VRE*fm* BSI risk, and carbapenems, BL/BLIs, and systemic antifungals were independently associated with VRE*fm* BSI. In the broader LBMT cohort, VRE*fm* BSI was associated with increased mortality.

Our high-risk composite served as a pragmatic measure of host vulnerability in this mixed LBMT cohort, given the limited sample size of individual primary diagnosis and transplant strata. This composite classification grouped patients with AML and/or allo-SCT recipients due to their shared biological and epidemiological characteristics [2, 10, 18, 23]. In a deconstructed sensitivity analysis, allo-SCT remained associated with VRE*fm* BSI, whereas AML lost statistical significance despite a stable point estimate. This pattern indicates inflated standard error and reduced precision rather than a definitive attenuation of the underlying association. Nonetheless, this composite should be regarded as a practical risk classification rather than conclusive evidence that AML and/or allo-SCT possess interchangeable or identical effects.

The association between AML and/or allo-SCT and VRE*fm* BSI is clinically plausible; complications of allo-SCT and AML chemotherapy include severe immunosuppression, prolonged neutropenia, and chemotherapy-induced mucosal injury, which increase susceptibility to bacterial translocation [2, 6, 13, 14, 24]. In contrast, auto-SCT recipients in our study exhibited a lower risk profile, supporting their exclusion from the high-risk composite. This disparity is evident across the SCT literature; a large series of auto-SCT recipients demonstrated that VRE colonization is common, yet progression to VRE BSI is infrequent, and survival is rarely impacted, in contrast to the risks observed in allo-SCT settings [3, 12]. Taken together, these findings highlight the heterogeneity of SCT-associated VRE risk and suggest that host susceptibility is required for antimicrobial and ecological pressures to drive progression to VRE*fm* BSI.

In cohorts with hematologic malignancy, the existing literature has identified several antimicrobial classes associated with VRE BSI [13, 25]. Mioljevic et al reported duration effects of piperacillin-tazobactam and exposure risk to carbapenems on VRE colonization outcomes in a cohort of patients admitted to the ICU with an underlying hematological malignancy [22]. This aligns with pharmacoepidemiology studies, showing that prolonged antibiotic courses increase VRE stool density and the risk of subsequent VRE BSI [26, 27]. In contrast to previous analyses, our assessment incorporated the cumulative antibiotic exposure alongside RCS models and demonstrated a duration-dependent association, with longer durations increasing the risk of VRE*fm* BSI for carbapenems, BL/BLIs, antianaerobic agents, and systemic antifungals. Although odds estimates appeared higher with longer antibiotic use, they became increasingly imprecise beyond approximately 15 days due to limited observations at higher exposure durations; as a result, no definitive exposure thresholds can be determined. Nonetheless, these findings highlight the potential importance of stewardship strategies rather than suggesting that a specific treatment duration will prevent VRE*fm* BSI [13, 22]. Published implementation studies in febrile neutropenia and leukemia cohorts confirm that carbapenem-sparing protocols reduce the hazard of VRE colonization by up to 65% and alter unit-level ecology, demonstrating that judicious antimicrobial use can attenuate selective pressure in high-risk populations [28]. Carbapenems, BL/BLIs, and antianaerobic agents converge on antianaerobic activity, which is associated with intestinal microbiome disruption. This disruption increases the risk of VRE*fm* BSI by suppressing protective obligate anaerobes and reducing intestinal colonization resistance, thereby facilitating VRE domination and subsequent translocation into the circulation [29]. In our study, we defined antianaerobic agents by spectrum, which overlapped with carbapenems and BL/BLIs spectrum. As a result, we excluded this class from the primary model; however, a separate RCS analysis of antianaerobic agents demonstrated a nonlinear association with VRE*fm* BSI.

Contextualizing these findings requires distinguishing causal risk factors and temporally associated exposures. Given the limitations of retrospective observational designs, cumulative antibiotic exposure should be interpreted as a potentially modifiable factor of a sustained antibiotic selection pressure in a high-risk setting, rather than an independently established causal exposure. Although the primary multivariable model adjusted for markers of clinical acuity, residual confounding by treatment indication and overall illness severity cannot be entirely excluded. We did not use hospital LOS as a matching variable since it is closely linked to illness severity and longer antibiotic exposure opportunity; matching on hospital LOS could therefore reduce clinically relevant variation in the exposure and potentially obscure associations between cumulative antibiotic exposure and VRE*fm* BSI. This phenomenon was reflected in the antibiotic exposure-density analysis, in which antibiotic class associations were no longer statistically significant, illustrating that the associations were sensitive to how exposure opportunity was represented and highlighting the preventable harm associated with prolonged antibiotic use in this population [14, 30].

While mortality remained stable across the overall LBMT cohort, VRE*fm* BSI-associated mortality increased. This trend should be interpreted with caution, as it may reflect temporal shifts in clinical acuity, antimicrobial resistance, and management. Furthermore, because our survival analysis evaluated all-cause rather than VRE*fm* BSI-attributable mortality, these findings do not establish direct causality. Nevertheless, a significant survival divergence persisted across both the 1- and 3-year follow-up windows, suggesting that VRE*fm* BSI may serve as a harbinger of profound host vulnerability in both the early phase and over the long term.

Our study has several methodological strengths. The matched case-control design reduced confounding and minimized variation inherent in single-center analyses. To our knowledge, this is one of the largest single-center studies evaluating factors associated with VRE*fm* BSI in populations with hematologic malignancy and in recipients of SCT [6, 13, 23, 31–33]. A key methodological strength is modeling cumulative antibiotic exposure using RCS, which enabled us to capture nonlinear relationships and visualize how marginal risks vary across the exposure distribution. Our analysis also indicates a unified high-risk composite across SCT and hematologic malignancy cohorts in a mixed-unit setting, highlighting its potential role in increasing VRE*fm* BSI risk. While allo-SCT appears to be the primary risk factor, the influence of specific malignancy subtypes–particularly AML–could not be fully disentangled due to overlap between these populations. Furthermore, because relevant clinical data such as post-transplant complications were not fully classifiable in our study cohort, the observed association of allo-SCT with VRE*fm* BSI should not be considered in isolation from post-transplant course complications. Larger studies with granular data are needed to determine whether specific allo-SCT-related complications, such as GVHD, drive VRE*fm* BSI risk.

Several limitations warrant acknowledgement. The retrospective, single-center design limits generalizability given differences in VRE epidemiology and antimicrobial prescribing practices across different healthcare settings. Additionally, the small number of VRE*fm* BSI among auto-SCT and NHL necessitated composite risk categorization to maintain model stability, potentially obscuring within-group heterogeneity. Furthermore, we acknowledge potential quasi-complete separation for severe neutropenia due to sparse data, which inflated standard errors in our multivariable models and led to fluctuating significance across sensitivity analyses. Notably, VRE colonization data were unavailable for much of the study period after routine screening was discontinued in 2018, precluding adjustment for colonization pressure. Due to limitations in our EMR data, we could not classify clinical data on the post-transplant course among SCT recipients, adjudicate the clinical indication for antimicrobial therapy, determine the status of the primary malignancy diagnosis, or measure other cumulative therapies that may introduce residual confounding. Moreover, although matching on admission indication and admission period reduced differences in admission context, it did not fully control for early versus late post-SCT status or newly diagnosed versus relapsed or refractory malignancy; therefore, the findings should be considered in the context of the overall hospitalized LBMT population rather than a specific post-transplant phase or disease status. Finally, although the incidence of VRE*fm* BSI increased over the study period, discontinuation of surveillance limited our ability to evaluate whether changes in infection control practices contributed to this trend.

## CONCLUSION

Our study demonstrates a duration-dependent, nonlinear association between cumulative exposure to select antibiotic classes and the risk of VRE*fm* BSI, particularly among patients with AML and/or recipients of allo-SCT. These findings highlight antimicrobial stewardship, specifically in minimizing unnecessary antibiotic duration, as a potentially modifiable clinical strategy that may reduce the burden of VRE*fm* BSI within this population.

## Supplementary Material

ofag627_Supplementary_Data

## Supplementary Data

Supplementary materials are available at *Open Forum Infectious Diseases* online. Consisting of data provided by the authors to benefit the reader, the posted materials are not copyedited and are the sole responsibility of the authors, so questions or comments should be addressed to the corresponding author.
