# Supplementary material for: Risk Factors and Outcomes of Vancomycin-Resistant Enterococcus faecium Bloodstream Infection in Hematologic Malignancies and Stem Cell Transplant Recipients: A Case-Control Study
Source: Open Forum Infect Dis. 2026 Oct 6;13(10):ofag627. doi: 10.1093/ofid/ofag627 (PMC13675249; doi:10.1093/ofid/ofag627)
Supplement: Supplementary file 1 [file ofag627_supplementary_data.docx]

**Supplementary Material**

Supplement to: Aldhaheri K, Fung J, Nishi C, et al. Risk Factors and Outcomes of Vancomycin-Resistant *Enterococcus faecium* Bloodstream Infection in Hematologic Malignancies and Stem Cell Transplant Recipients: A Case-Control Study

**Table of Contents**

1. **Definitions** .................................................................................................................**3**
2. **Table S1.** Sensitivity analysis: factors associated with VRE*fm* BSI by primary diagnosis or SCT type………………………………………..……………………....…….**5**
3. **Table S2.** Distribution of SCT by primary hematological malignancy diagnosis ….…**6**
4. **Table S3.** factors associated with VRE*fm* BSI by primary diagnosis or SCT type along with other factors………………………………….………...……………………………….**7**
5. **Table S4.** Sensitivity analysis of antibiotic exposure density and risk of VRE*fm* BSI..**8**
6. **Figure S1. Kaplan-Meier survival curves for 3-year overall survival in the overall LBMT cohort………………………………………………………………………………...9**

**Definitions**

- **Hospitalized patient with VRE*fm* BSI:** Patient meeting inclusion criteria whose date of admission to the hospital precedes the date of the first positive VRE*fm* BSI blood culture.
- **Outpatient with VRE*fm* BSI:** Patient whose admission date is subsequent to the date of the first positive VRE*fm* blood culture.
- **Matching criteria**
  - Assessment: All first-time admissions to the Vancouver General Hospital LBMT unit were labelled as assessment admissions regardless of the reason for admission.
  - Chemotherapy: Elective admissions to receive chemotherapy treatment.
  - Transplant: Elective admissions for stem cell transplant.
  - Complications: Admissions due to any other reason
- **Antimicrobials:**
  - Beta-lactams: Oral or parenteral amoxicillin-clavulanate, ticarcillin-clavulanate, penicillin, amoxicillin, ampicillin, piperacillin-tazobactam, meropenem, imipenem-cilastatin, ertapenem, cephalosporins.
  - Beta-lactams/beta-lactamase inhibitors: Oral or parenteral amoxicillin-clavulanate, ticarcillin-clavulanate, piperacillin-tazobactam.
  - Cephalosporins: Oral or parenteral agents of any class of cephalosporins.
  - Carbapenems: Parenteral meropenem, imipenem-cilastatin, ertapenem.
  - Systemic antifungals: Oral or parenteral fluconazole, voriconazole, posaconazole, isavuconazole, itraconazole, micafungin, caspofungin, amphotericin.
  - Anti-anaerobic agents: Oral or parenteral amoxicillin-clavulanate, piperacillin-tazobactam, meropenem, imipenem-cilastatin, ertapenem, metronidazole, clindamycin, moxifloxacin.
  - Vancomycin: parenteral and/or oral vancomycin.
  - Fluoroquinolones: Oral or parenteral moxifloxacin, levofloxacin, ciprofloxacin.
- **Time at risk period:**
  - Cases: From the date of hospital admission to the date of onset of VRE*fm* BSI (index date).
  - Controls: Assigned the matched case’s index date when feasible by applying the same interval from their admission date, or discharge date if the control left the hospital earlier.
- **Hospital LOS:**
  - Calculated in days from admission date to discharge date.
- **ICU admission:**
  - Admission to ICU during hospitalization at any point during hospital LOS.
- **Severe neutropenia:**
  - Defined as an absolute neutrophil count <0.5 x10^9^/L at any point during hospital LOS.

| **Primary diagnosis or transplant type** | **Crude OR (95% CI)** | ***p*-value** | **Adjusted OR (95% CI)** | ***p*-value** |
| --- | --- | --- | --- | --- |
| Allogeneic SCT  Autologous SCT | 31.05 (7.27–132.73)  0.03 (0.004–0.21) | <0.001  0.001 | 20.65 (2.65–176.68)  0.68 (0.03–9.75) | 0.006  0.799 |
| AML | 2.37 (1.45–3.88) | 0.001 | 1.99 (1.34–4.49) | 0.031 |
| ALL | 1.32 (0.74–2.35) | 0.352 | 1.19 (0.86–3.74) | 0.660 |
| NHL | 0.26 (0.12–0.59) | 0.001 | 0.96 (0.17–1.07) | 0.938 |
| HL | 0.58 (0.12–2.78) | 0.492 |  |  |
| MPD/MDS | 1.36 (0.69–2.68) | 0.378 |  |  |

**Table S1**. Sensitivity analysis of primary diagnosis and SCT type: unadjusted and adjusted odds ratios of primary diagnosis or transplant category associated with VRE*fm* BSI in the matched case-control population (n=500)

Recipients of SCT were classified by SCT type if transplanted, or by primary hematologic malignancy diagnosis.

Abbreviation: VRE*fm*: vancomycin-resistant *Enterococcus faecium*, BSI: bloodstream infection, OR: odds ratio, CI: confidence interval, SCT: stem cell transplant, allo-SCT: allogeneic stem cell transplant, AML: acute myeloid leukemia, ALL: acute lymphoblastic leukemia, NHL: non-Hodgkin lymphoma, HL: Hodgkin lymphoma, MPD: myeloproliferative disorder, MDS: myelodysplastic syndrome

**Table S2**. Distribution of stem cell transplant by primary hematological malignancy diagnosis in the matched case-control population (n=500)

| **Primary diagnosis** | **Total** | **Allo-SCT, N=93** | **Auto-SCT, N=69** | **Non-SCT, N=338** |
| --- | --- | --- | --- | --- |
| AML | 199 (39.8) | 33 (35.5) | 0 | 166 (33.2) |
| ALL | 75 (15.0) | 20 (21.5) | 1 (1.5) | 54 (10.8) |
| NHL | 90 (18.0) | 12 (12.9) | 45 (65.2) | 33 (6.6) |
| Other^1^ | 136 (27.2) | 28 (30.1) | 23 (33.3) | 85 (17.0) |

^1^Hodgkin lymphoma, myeloproliferative disorder, aplastic anemia, multiple myeloma, other leukemia, and other

Abbreviations: SCT: stem cell transplant, allo-SCT: allogeneic stem cell transplant, auto-SCT: autologous stem cell transplant, AML: acute myeloid leukemia, ALL: acute lymphoblastic leukemia, NHL: non-Hodgkin lymphoma.

**Table S3.** Sensitivity analysis of primary diagnosis or SCT type: Unadjusted and adjusted odds ratios for factors associated with VRE*fm* BSI in the matched case-control population (n=500)

| **Variable** | **Crude OR (95% CI)** | ***p*-value** | **Adjusted OR (95% CI)** | ***p*-value** |
| --- | --- | --- | --- | --- |
| Allogenic SCT  Autologous SCT | 31.05 (7.27–132.73)  0.03 (0.004–0.21) | <0.001  0.001 | 17.48 (1.58–193.04)  1.21 (0.04–34.57) | 0.020  0.912 |
| AML | 2.37 (1.45–3.88) | 0.001 | 2.02 (0.86–4.71) | 0.105 |
| ALL | 1.32 (0.74–2.35) | 0.352 | 1.27 (0.40–4.08) | 0.683 |
| NHL | 0.26 (0.12–0.59) | 0.001 | 0.95 (0.25–3.63) | 0.935 |
| Severe neutropenia | 14.23 (3.39–59.78) | <0.001 | 5.66 (0.76–42.02) | 0.090 |
| ICU admission | 14.14 (7.04–28.38) | <0.001 | 6.97 (2.47–19.65) | <0.001 |
| Hospital LOS | 1.05 (1.03–1.06) | <0.001 | 1.01 (0.99–1.02) | 0.459 |
| Carbapenem | 1.13 (1.08–1.17) | <0.001 | 1.09 (1.01–1.17) | 0.026 |
| BL-BLI | 1.10 (1.06–1.14) | <0.001 | 1.08 (1.02–1.15) | 0.014 |
| Vancomycin | 1.14 (1.08–1.20) | <0.001 | 0.96 (0.88–1.05) | 0.413 |
| Fluoroquinolone | 1.16 (1.07–1.25) | <0.001 | 1.23 (1.03–1.23) | 0.012 |
| Systemic antifungals | 1.12 (1.14–1.30) | <0.001 | 1.12 (1.04–1.24) | 0.007 |

Abbreviations: VRE*fm*: vancomycin-resistant *Enterococcus faecium*, BSI: bloodstream infection, SCT: stem cell transplant, AML: acute myeloid leukemia, ALL: acute lymphoblastic leukemia, NHL: non-Hodgkin lymphoma, ICU: intensive care unit, LOS: length of stay, BL-BLIs inhibitors: beta-lactam/beta-lactamase inhibitors, OR: odds ratio, CI: confidence interval.

**Table S4.** Sensitivity analysis of antibiotic exposure density: Unadjusted and adjusted odds ratios for factors associated with VRE*fm* BSI in the matched case-control population (n=500)

| **Variable** | **Crude OR (95% CI)** | ***p*-value** | **Adjusted OR (95% CI)** | ***p*-value** |
| --- | --- | --- | --- | --- |
| High-risk status^1^ | 4.38 (2.60–7.41) | <0.001 | 2.66 (1.37–5.13) | 0.004 |
| Severe neutropenia | 14.23 (3.39–59.78) | <0.001 | 9.56 (1.41–64.90) | 0.021 |
| Hospital length of stay | 1.05 (1.03–1.06) | <0.001 | 1.03 (1.01–1.04) | <0.001 |
| ICU admission | 14.14 (7.04–28.38) | <0.001 | 6.95 (3.01–16.02) | <0.001 |
| BL/BLIs | 1.13 (1.08–1.17) | <0.001 | 1.20 (0.29–5.07) | 0.802 |
| Carbapenem | 1.10 (1.06–1.14) | <0.001 | 1.74 (0.29–10.44) | 0.547 |
| Fluoroquinolones | 1.16 (1.07–1.25) | <0.001 | 0.38 (0.02–7.47) | 0.524 |
| Vancomycin | 1.14 (1.08–1.20) | <0.001 | 0.32 (0.03–3.69) | 0.360 |
| Systemic antifungal | 1.12 (1.14–1.30) | <0.001 | 1.18 (0.28–4.91) | 0.819 |

^1^High-risk status: AML and/or allo-SCT regardless of indication

Antibiotic exposure density was defined as days of therapy for each antibiotic class divided by length of stay during the risk period.

Abbreviations: VRE*fm*: vancomycin-resistant *Enterococcus faecium*, BSI: bloodstream infection, OR: odds ratio, CI: confidence interval, ICU: intensive care unit, BL-BLIs: beta-lactam/beta-lactamase inhibitors.

**Figure S1**. Kaplan-Meier survival curves for 3-year overall survival in the overall LBMT cohort

Abbreviations: VRE: vancomycin-resistant *Enterococcus faecium*, BSI: bloodstream infection, LBMT: leukemia and bone marrow transplant
